# Diffusible Signal Factors Act through AraC-Type Transcriptional Regulators as Chemical Cues To Repress Virulence of Enteric Pathogens

**DOI:** 10.1128/IAI.00226-20

**Published:** 2020-09-18

**Authors:** Erick Maosa Bosire, Colleen R. Eade, Carl J. Schiltz, Amanda J. Reid, Jerry Troutman, Joshua S. Chappie, Craig Altier

**Affiliations:** a Department of Population Medicine and Diagnostic Sciences, College of Veterinary Medicine, Cornell University, Ithaca, New York, USA; b Department of Chemistry, University of North Carolina at Charlotte, Charlotte, North Carolina, USA; c Department of Molecular Medicine, College of Veterinary Medicine, Cornell University, Ithaca, New York, USA; Stanford University

**Keywords:** diffusible signal factors, fatty acids, gene expression, host cell invasion, transcriptional regulation, virulence regulation

## Abstract

Successful colonization by enteric pathogens is contingent upon effective interactions with the host and the resident microbiota. These pathogens thus respond to and integrate myriad signals to control virulence. Long-chain fatty acids repress the virulence of the important enteric pathogens Salmonella enterica and Vibrio cholerae by repressing AraC-type transcriptional regulators in pathogenicity islands. While several fatty acids are known to be repressive, we show here that *cis*-2-unsaturated fatty acids, a rare chemical class used as diffusible signal factors (DSFs), are highly potent inhibitors of virulence functions. We found that DSFs repressed virulence gene expression of enteric pathogens by interacting with transcriptional regulators of the AraC family. In Salmonella enterica serovar Typhimurium, DSFs repress the activity of HilD, an AraC-type activator essential to the induction of epithelial cell invasion, by both preventing its interaction with target DNA and inducing its rapid degradation by Lon protease. *cis*-2-Hexadecenoic acid (c2-HDA), a DSF produced by Xylella fastidiosa, was the most potent among those tested, repressing the HilD-dependent transcriptional regulator *hilA* and the type III secretion effector *sopB* >200- and 68-fold, respectively. Further, c2-HDA attenuated the transcription of the ToxT-dependent cholera toxin synthesis genes of V. cholerae. c2-HDA significantly repressed invasion gene expression by *Salmonella* in the murine colitis model, indicating that the HilD-dependent signaling pathway functions within the complex milieu of the animal intestine. These data argue that enteric pathogens respond to DSFs as interspecies signals to identify appropriate niches in the gut for virulence activation, which could be exploited to control the virulence of enteric pathogens.

## Abstract (precis)

Successful colonization by enteric pathogens is contingent upon effective interactions with the host and the resident microbiota. These pathogens thus respond to and integrate myriad signals to control virulence. Long-chain fatty acids repress the virulence of the important enteric pathogens Salmonella enterica and Vibrio cholerae by repressing AraC-type transcriptional regulators in pathogenicity islands. While several fatty acids are known to be repressive, we show here that *cis*-2-unsaturated fatty acids, a rare chemical class used as diffusible signal factors (DSFs), are highly potent inhibitors of virulence functions.

## INTRODUCTION

In the intestinal milieu, pathogens engage in intricate interactions with the host and the microbiota that often lead to pathogen colonization resistance (1–3). To penetrate this colonization barrier, enteric pathogens regulate their virulence in response to gut environmental factors to ensure a timely activation and minimization of fitness costs (4). Many pathogens therefore integrate a multitude of host and environmental signals with metabolic cues to optimize their virulence generation pathways (5–8). Many of these cues converge at the central transcriptional regulators of the AraC family in pathogenicity islands (5, 7, 9, 10).

In *Salmonella*, the type III secretion system encoded by genes in *Salmonella* pathogenicity island 1 (SPI1) is controlled by the AraC-type transcriptional regulator HilD (11). HilD forms a feed-forward loop with AraC family members HilC and RtsA to induce *hilA* (see Fig. S1 in the supplemental material) (12). HilA, in turn, activates the expression of genes encoding the needle complex and secreted effector proteins for invasion of epithelial cells (13). AraC family transcriptional regulators control virulence mechanisms in several pathogens, including type III secretion in Shigella flexneri (VirF) and Yersinia pestis (LcrF) and adhesion fimbriae in enterotoxigenic Escherichia coli (Rns) (14). In Vibrio cholerae, the AraC-type transcriptional regulator ToxT regulates genes encoding the virulence factors in the *Vibrio* pathogenicity island (VPI) (15). ToxT functions as the master regulator integrating environmental signals to control genes encoding cholera toxin (*ctxAB*) and toxin-coregulated pilus (*tcpA*) (16).

Short- and long-chain fatty acids produced by the host and microbiota regulate virulence of the important enteric pathogens *Salmonella* and V. cholerae by interacting with transcriptional regulators of the AraC family (8, 9, 17–20). Butyric acid and propionic acid, which exist in high concentrations in the gut, and oleic acid, which is abundant in bile, have been shown to regulate SPI1 through HilD (8, 9, 17). These fatty acids are host and microbiota derived as dietary fatty acids are highly absorbed in the upper intestinal tract (21). In V. cholerae, unsaturated fatty acids present in bile repress virulence by interacting with the HilD homolog ToxT (19). While these transcriptional regulators have been shown to accommodate different sizes of fatty acids *in vitro* (9, 19, 20), the specific fatty acid repressors in the gut have not been identified.

A rare class of *cis*-2-unsaturated fatty acids is used by several bacterial pathogens of animals and plants to regulate quorum sensing-dependent behaviors such as biofilm formation (22). Termed diffusible signal factors (DSFs), these include molecules with various chain lengths and substituents. *cis*-11-Methyl-2-dodecenoic acid was the first to be characterized from Xanthomonas campestris and later in Stenotrophomonas maltophilia (23); others shown to influence pathogenicity include *cis*-2-hexadecenoic acid (c2-HDA), *cis*-2-decenoic acid, and *cis*-2-dodecenoic acid, produced by Xylella fastidiosa, Pseudomonas aeruginosa, and Burkholderia cenocepacia, respectively (22, 24–27). DSFs are produced by unique crotonases that contain both a 3-hydroxyacyl-acyl carrier protein (ACP) dehydratase and an esterase activity (28). Signal recognition and transduction occur differently among the species that produce them. In X. fastidiosa, DSFs are recognized through the outer membrane sensor kinase RpfC, which phosphorylates the phosphodiesterase regulator RpfG (29, 30). In B. cenocepacia, however, DSFs are recognized by the cytoplasmic GGDEF-EAL domain protein RpfR, which shows phosphodiesterase activity (31). Both pathways regulate cyclic di-GMP turnover, which, in turn, regulates genes responsible for virulence and adaptation (22, 30). Different species produce and respond to varied chain lengths, and cross-species activity of DSFs has been reported for several plant and animal pathogens (22, 27).

Here we show that the DSF c2-HDA is a highly potent inhibitor of enteric pathogen virulence gene expression. c2-HDA acts by interacting with the central transcriptional regulators of SPI1, and most likely the VPI, both of which are required for successful gut colonization (32–34).

## RESULTS

### The diffusible signal factor c2-HDA is a highly potent inhibitor of virulence gene expression.

**FIG 1:**
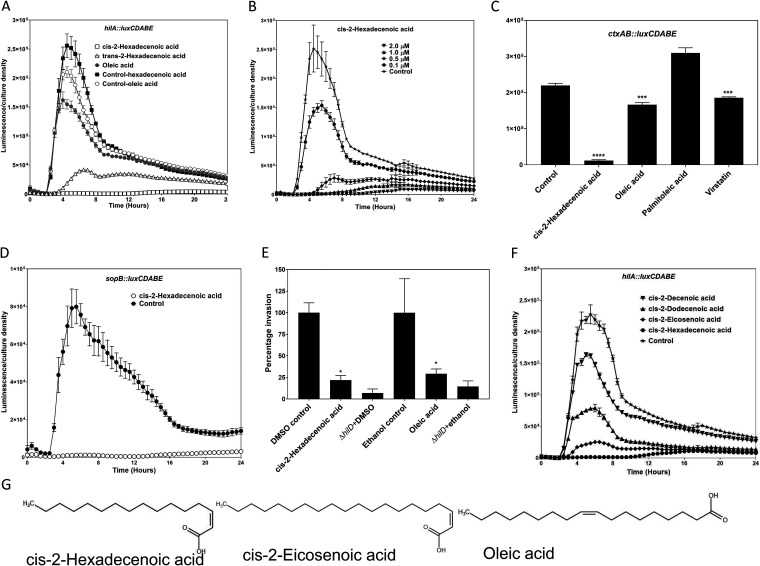
The DSF *cis*-2-hexadecenoic acid potently represses virulence expression. (A) *cis*-2-hexadecenoic acid inhibits *Salmonella hilA* expression, while its *trans*-isomer is less potent. A strain carrying a *hilA*::*luxCDABE* reporter plasmid was grown in the presence of 5 μM fatty acids. The control cultures contained the vehicle only (dimethyl sulfoxide [DMSO] for *cis*-2-hexadecenoic acid and *cis*-2-eicosenoic acid and ethanol for oleic acid) at a concentration identical to that of the treated culture. (B) *cis*-2-hexadecenoic acid potently represses *hilA* expression at low concentrations. (C) The DSF represses *Vibrio ctxAB* genes encoding the cholera toxin when supplied at 20 μM. (D) The DSF potently represses the *Salmonella* type III secretion complex effector protein gene *sopB*. A strain carrying a *sopB*::*luxCDABE* reporter plasmid was grown in the presence of 20 μM *cis*-2-hexadecenoic acid. (E) The DSF reduces HEp-2 cell invasion by *Salmonella*. The number of bacteria that invaded HEp-2 cells in the presence of the DSF was determined using a gentamicin protection assay. (F) *cis*-2-hexadecenoic acid contains the effective chain length for repressing *hilA*. (G) Structures of *cis*-2-hexadecenoic acid and the controls *cis*-2-eicosenoic acid and oleic acid. Expression of *lux* reporter fusions is presented as luminescence normalized to bacterial culture density. Error bars represent standard deviations of 5 replicates for panels A, B, D, and F, 3 for panel C, and 4 for panel E. The control culture contained the vehicle only at a concentration identical to that of the chemical-containing cultures. Asterisks indicate expression levels significantly different from that of the control (****, *P* < 0.0001; ***, *P* < 0.001; *, *P* < 0.05).

In *Salmonella*, long- and short-chain fatty acids repress the invasion genes of SPI1 through the posttranscriptional control of the central transcriptional regulator HilD (9, 17, 18). We sought to identify related chemicals that can potently inhibit invasion gene expression and determine the mechanisms by which they repress these genes. We tested the efficacy of a rare class of fatty acids with a characteristic *cis*-2 unsaturation, termed DSFs (22, 24, 26, 27). A *Salmonella* strain carrying a *hilA*::*luxCDABE* reporter fusion was used to monitor the effects of *cis*-2-unsaturated fatty acids on SPI1-encoded invasion gene expression, as HilA directly activates expression of genes responsible for the production of the type III secretion complex and effector proteins (6, 13, 35). When supplied to cultures at a concentration of 5 μM, c2-HDA significantly repressed *hilA* expression (>200-fold), to a level that was undetectable in our assay. For comparison, oleic acid, which has been shown to repress SPI1 through its effects on HilD (9), slightly repressed *hilA* (1.3-fold) at the same concentration (Fig. 1A). This chemical did not impair bacterial growth (Fig. S1B). Moreover, c2-HDA proved to maintain its potency at a range of concentrations, repressing 80-fold at 1 μM and significantly inhibiting *hilA* expression (39%) at 100 nM (Fig. 1B). We next tested whether c2-HDA regulated the virulence of V. cholerae. Unlike *Salmonella*, V. cholerae is noninvasive, but it requires the production of cholera toxin for colonization (36). Fatty acids repress the virulence of this pathogen by binding to ToxT, the transcriptional activator of the cholera toxin genes *ctxAB* (19, 20, 37). Using a *ctxAB*::*luxCDABE* fusion, we found that c2-HDA significantly repressed *ctxAB* (20-fold). In comparison, oleic acid and the small-molecule inhibitor virstatin, both known to repress ToxT, slightly repressed *ctxAB* (1.3- and 1.2-fold, respectively), while palmitic acid had no repressive effects at the same concentration (Fig. 1C).

We further investigated the mechanisms by which DSFs repress virulence in *Salmonella*. We first tested whether c2-HDA repressed genes encoding type III secretion effector proteins using a *sopB*::*luxCDABE* reporter fusion, as the effector protein SopB is essential for invasion of epithelial cells (38). c2-HDA significantly repressed *sopB* expression (68-fold) (Fig. 1D). These data suggest that the repression of SPI1 by c2-HDA leads to transcriptional inhibition of effector protein genes. We thus next tested the invasion competency of bacteria grown in the presence of the c2-HDA. Overnight growth of *Salmonella* in the presence of c2-HDA significantly decreased its invasion of HEp-2 cells (by 78%) compared to untreated cultures, while oleic acid reduced invasion by 70% at the same concentration (Fig. 1E). Together, these data demonstrate that c2-HDA represses invasion gene expression and the ability of *Salmonella* to invade epithelial cells.

**FIG 2:**
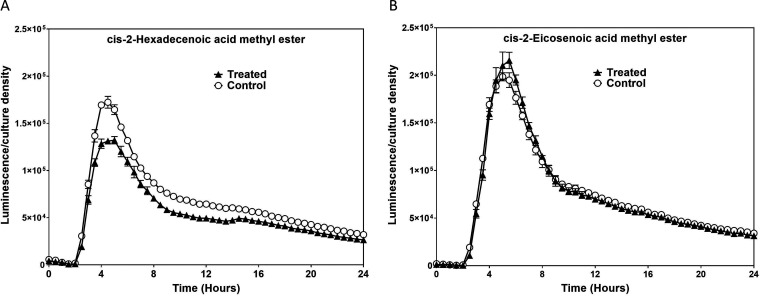
Methylation of the carboxyl end reduces the potency of *cis*-2-unsaturated fatty acids. A strain carrying a *hilA*::*lux* reporter plasmid was grown in the presence of 40 μM *cis*-2-eicosenoic acid methyl ester (A) and 20 μM *cis*-2-hexadecenoic acid methyl ester (B). Expression of *hilA* is reported as mean luminescence normalized to bacterial culture density. Error bars represent standard deviations of 5 replicates. The control culture contained the vehicle only at a concentration identical to that of the treated culture.

The *cis*-2 unsaturation of DSFs is the essential signature for quorum signaling, as *trans*-2-unsaturated isomers elicit minimal effects (27). We therefore tested the potency of *trans*-2-hexadecenoic acid in repressing *hilA*. The *trans*-isomer was 31-fold less potent in repressing *hilA* than was the *cis*-isomer, indicating a specificity of the *cis*-2 unsaturation orientation (Fig. 1A). We next examined how the chain length affected the repressive properties of *cis*-2-unsaturated compounds. Among the DSFs tested, the 16-carbon c2-HDA was the most potent, significantly reducing *hilA* expression (159-fold) (Fig. 1F). The 12-carbon DSF *cis*-2-dodecenoic acid also significantly reduced *hilA* expression, but to a much lesser extent, 3-fold. The least potent was the 10-carbon *cis*-2-decenoic acid, which slightly reduced *hilA* expression (by 28%) (Fig. 1F). Additionally, the 20-carbon *cis*-2-eicosenoic acid, unknown as a DSF but differing from recognized DSFs by only its length, repressed *hilA* 10-fold (Fig. 1F and G). These comparisons indicate that chain length, saturation, and bond geometry all influence the repressive properties and potency of DSFs with regard to AraC transcriptional regulators. We also determined whether the carboxyl end of the fatty acids played any role in the repression of invasion genes. Methyl esters of c2-HDA and *cis*-2-eicosenoic acid showed reduced potency in repressing *hilA* expression, indicating the importance of the terminal carboxyl group for the activity of these *cis*-2-unsaturated fatty acids (Fig. 2).

**FIG 3:**
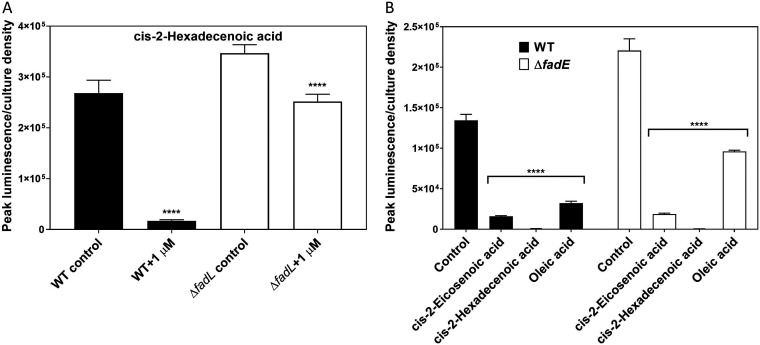
Repressive effects of *cis*-2-hexadecenoic acid are dependent on the fatty acid transporter but independent of β-oxidation. (A) The DSF represses *hilA* less potently in the absence of the long-chain fatty acid transporter FadL. A Δ*fadL* mutant carrying a *hilA*::*luxCDABE* reporter plasmid was grown in the presence of 1 μM DSF. (B) A Δ*fadE* mutant carrying a *hilA*::*lux* reporter fusion was grown in the presence of 20 μM *cis*-2-unsaturated fatty acids. Expression of *hilA* is presented as peak luminescence normalized to bacterial culture density. Error bars represent standard deviations of 5 replicates. The control culture contained the vehicle only at a concentration identical to that of the chemical-containing cultures. Asterisks indicate expression levels significantly different from that of the control (****, *P* < 0.0001). WT, wild type.

Long-chain fatty acids are transported actively by the long-chain fatty acid transporter FadL (39). We next tested whether c2-HDA continued to repress *hilA* in the absence of FadL. In a *fadL* null mutant, 1 μM c2-HDA was 39% less potent in repressing *hilA* than the wild type (Fig. 3A). These data suggest that FadL-mediated transport is essential for the activity of *cis*-2-unsaturated fatty acids, as has been reported for oleic acid (9).

The results described above suggest that a precise chemical structure is necessary for the activity of *cis*-2-unsaturated fatty acids on SPI1 virulence genes. We therefore hypothesized that these compounds repress directly, rather than through degradation products. To test this, we disrupted the β-oxidation pathway, by which fatty acid compounds are degraded. FadE is an acyl coenzyme A (acyl-CoA) dehydrogenase that catalyzes the first step of β-oxidation, converting acyl-CoA to 2-enoyl-CoA (40). As has been reported for oleic acid, *cis*-2-unsaturated fatty acids continued to repress *hilA* in the absence of *fadE*, suggesting that their effects are independent of degradation *via* β-oxidation (Fig. 3B).

### *cis*-2-unsaturated fatty acids inhibit the transcription activator of invasion HilD.

**FIG 4:**
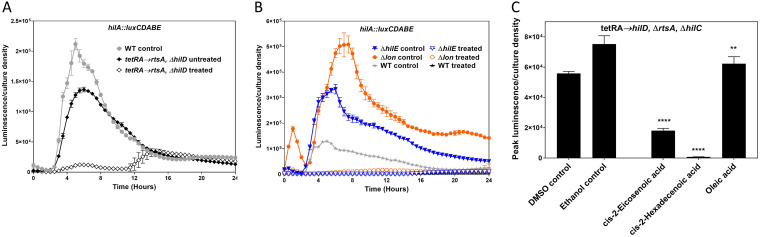
The DSF primarily targets the central SPI1 regulator HilD posttranscriptionally. (A) Loss of *hilD* reduces the repressive effects of *cis*-2-hexadecenoic acid on *sopB*. A Δ*hilD* mutant strain carrying a *sopB*::*lux* reporter fusion, and with *rtsA* under the control of a tetracycline-inducible promoter, was grown in the presence of 20 μM *cis*-2-hexadecenoic acid. (B) The DSFs’ repressive effects on *sopB* are independent of the HilD negative regulators HilE and Lon. Strains lacking *hilE* and *lon* and carrying a *sopB*::*lux* reporter fusion were grown in the presence of 20 μM *cis*-2-hexadecenoic acid. (C) *cis*-2-unsaturated fatty acids repress *hilD* posttranscriptionally. A strain lacking *rtsA* and *hilC*, and with *hilD* under the control of a tetracycline-inducible promoter and additionally carrying a *hilA-lux* reporter fusion, was grown in the presence of 20 μM *cis*-2-unsaturated fatty acids. A tetracycline concentration inducing *hilD* to a level equivalent to that of the wild type was used. Expression of *lux* reporter fusions is reported as mean luminescence normalized to bacterial culture density. Error bars represent standard deviations of 5 replicates. The control culture contained the vehicle only (DMSO for *cis*-2-hexadecenoic acid and *cis*-2-eicosenoic acid and ethanol for oleic acid) at a concentration identical to that of to the treated culture. Asterisks indicate expression levels significantly different from that of the control (****, *P* < 0.0001; **, *P* < 0.01).

In some bacteria, DSFs signal through two-component systems that utilize a transmembrane sensory kinase, and thus, the perception of the signals occurs extracellularly (22, 30). This raised the question of whether DSFs act extracellularly in *Salmonella* or whether they must instead be transported into the bacterial cytoplasm. HilD activates type III secretion complex genes, essential for invasion, both through and independent of *hilA* (12). Short- and long-chain fatty acids have also been shown to repress HilD activity (9, 17, 18). To elucidate the importance of HilD in repression by c2-HDA, we assessed the expression of *sopB* in a Δ*hilD* mutant in the presence of this chemical. *sopB* expression is low in the absence of *hilD*, reducing sensitivity of the luciferase assay. As *rtsA* modestly activates *sopB* transcription, sensitivity of the assay was improved by increasing expression of *rtsA* using a regulated tetracycline-inducible promoter (P*_tetRA_*) (41). c2-HDA repressed *sopB* 11-fold, compared to 68-fold in the wild type. This suggests that c2-HDA potentially represses SPI1 through multiple regulators (Fig. 1D and 4A). HilD is under the control of several regulators within and outside SPI1. It is downregulated by Lon protease (42) and HilE (43). As c2-HDA was repressive, we tested whether its effects were through these negative regulators. As may be expected, *sopB* expression was elevated in Δ*lon* and Δ*hilE* mutants (4-and 3-fold, respectively) compared to that in the wild type. Despite this increased expression, c2-HDA inhibited *sopB* expression in these mutants to the level observed in the wild-type strain (Fig. 4B). Hence, loss of these regulators had no effect on repression by c2-HDA. These results thus implicate HilD as one of the targets of c2-HDA, with additional effects independent of this regulator.

We next sought to determine whether these chemicals affect HilD directly and to elucidate the mechanisms of their repression. HilD forms part of a complex feed-forward loop with the transcriptional activators RtsA and HilC, which together induce *hilA* expression (41) (Fig. S1). To isolate the effects of *cis*-2-unsaturated fatty acids on HilD, *hilC* and *rtsA* were deleted, and a *hilA*::*luxCDABE* fusion was used to assess invasion gene expression. Because HilD controls its own transcription, we also replaced its native promoter with a tetracycline-inducible promoter. We first determined the concentration of tetracycline that induced *hilA* expression to a level equivalent to that of a wild type (5 μg/ml). Using this level of expression, we found that c2-HDA repressed *hilA* 78-fold, while *cis*-2-eicosenoic acid and oleic acid repressed less potently, 3- and 1.2-fold, respectively (Fig. 4C). As the expression of *hilD* is controlled in this strain, this result thus demonstrates that *cis*-2-unsaturated fatty acids function to repress invasion gene expression through their posttranscriptional modulation of HilD.

### *cis*-2-unsaturated fatty acids destabilize HilD.

**FIG 5:**
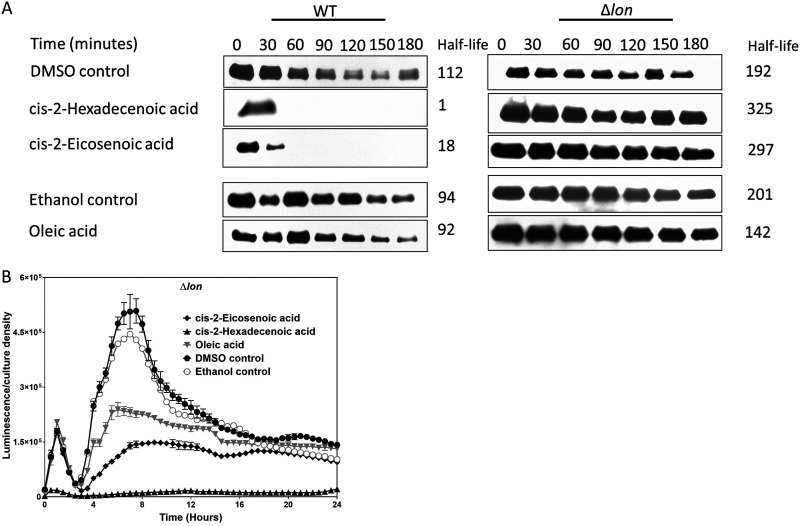
*cis*-2-unsaturated fatty acids inactivate HilD with consequent degradation by Lon. (A) *cis*-2-unsaturated fatty acids reduce HilD half-life in the presence of Lon. Strains carrying a *hilD*-3×FLAG construct under the control of a tetracycline-inducible promoter, with *lon* present or absent, were grown in the presence of 20 μM *cis*-2-unsaturated fatty acids. HilD half-life was determined by Western blotting for 3×FLAG. (B) *cis*-2-unsaturated fatty acids repress *hilA* expression in the absence of *lon*. A strain carrying a *hilA*::*lux* reporter fusion with a Δ*lon* mutation was grown in the presence of 20 μM fatty acids. Expression of *hilA* is presented as luminescence normalized to bacterial culture density. The control culture contained the vehicle only (DMSO for *cis*-2-hexadecenoic acid and *cis*-2-eicosenoic acid and ethanol for oleic acid) at a concentration identical to that of the treated culture.

To elucidate the possible mechanisms by which DSFs repressed *hilD* posttranscriptionally, we assessed their effects on HilD protein stability. A strain carrying *hilD* under the control of a tetracycline-inducible promoter and a C-terminal 3×FLAG tag was used to measure the stability of HilD. The half-life of HilD from bacteria grown in the absence of DSFs was 112 min, but addition of c2-HDA to the culture drastically reduced that half-life, to 1 min (Fig. 5A). Consistent with the invasion gene expression results described above, *cis*-2-eicosenoic acid reduced HilD half-life by a lesser extent, to 18 min, and oleic acid did so to 92 min (Fig. 5A). These data indicate that DSFs destabilize HilD, as we have previously reported for short-chain fatty acids and bile (7, 17). Lon protease is known to be responsible for HilD degradation (44), but our genetic approaches indicated that *lon* was not required for the repressive effects of c2-HDA (Fig. 4B). We therefore tested the role of Lon by assessing the HilD protein half-life in a *lon* mutant (44). In the absence of *lon*, HilD protein accumulated, and the DSF had no effect on its stability (Fig. 5A). However, the DSF continued to repress *hilA* expression even in the absence of *lon* (Fig. 5B). It is therefore likely that DSFs inactivate HilD with consequent degradation by Lon but that Lon plays no direct role in the repression of invasion genes by DSFs.

### *cis*-2-unsaturated fatty acids may target other SPI1 AraC transcriptional regulators.

**FIG 6:**
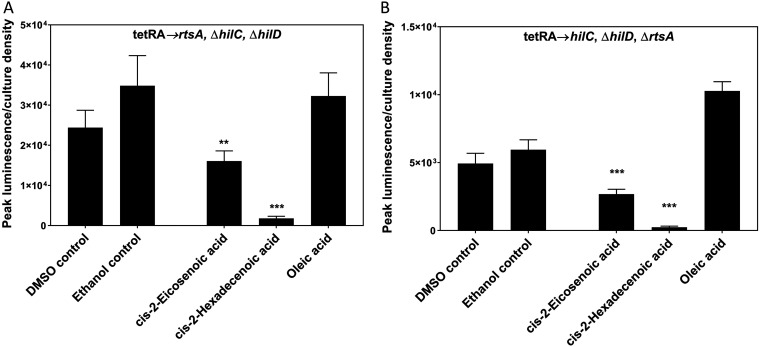
*cis*-2-unsaturated fatty acids may additionally repress other SPI1 transcriptional regulators of the AraC family. Strains carrying a *hilA*::*lux* reporter fusion, with either *rtsA* or *hilC* under the control of a tetracycline-inducible promoter and with null mutations of *hilD* and the remaining regulator (*rtsA* or *hilC*), were used. (A) *cis*-2-fatty acids repress *hilA* in the presence of *rtsA* only. (B) *cis*-unsaturated fatty acids repress *hilA* in the presence of *hilC* only. Expression of the *lux* reporter fusion is presented as peak luminescence normalized to bacterial culture density. The control culture contained the vehicle only (DMSO for *cis*-2-hexadecenoic acid and *cis*-2-eicosenoic acid and ethanol for oleic acid) at a concentration identical to that of the treated culture. Asterisks indicate expression levels significantly different from that of the control (***, *P* < 0.001; **, *P* < 0.01).

Our data show that HilD is important for the repressive effects of c2-HDA on invasion genes. In a *hilD* mutant, however, c2-HDA continued to demonstrate repression of *hilA* (Fig. 4A), suggesting the existence of additional means, independent of HilD, by which these compounds repress invasion. HilC and RtsA transcriptional regulators bind to the same promoters as does HilD (45, 46), and the three share 10% identity in their N termini (14). Based on these properties, we reasoned that HilC and RtsA might be additionally targeted by this compound. To test this, we utilized strains that express either *rtsA* or *hilC* under the control of a tetracycline-inducible promoter and also contain null mutations of *hilD* and the remaining regulator (*hilC* or *rtsA*). In the presence of only *hilC* or *rtsA*, c2-HDA significantly reduced *hilA* expression, 20- and 13-fold, respectively, compared to 78-fold in the presence of *hilD* only (Fig. 4C and Fig. 6). This suggests that c2-HDA may additionally target HilC and RtsA posttranscriptionally. Although these experiments clearly indicate that c2-HDA represses invasion through these three AraC-type regulators, we cannot rule out the possibility of effects through additional, as-yet-unidentified target proteins.

### *cis*-2-unsaturated fatty acids inhibit HilD from binding its target DNA.

**FIG 7:**
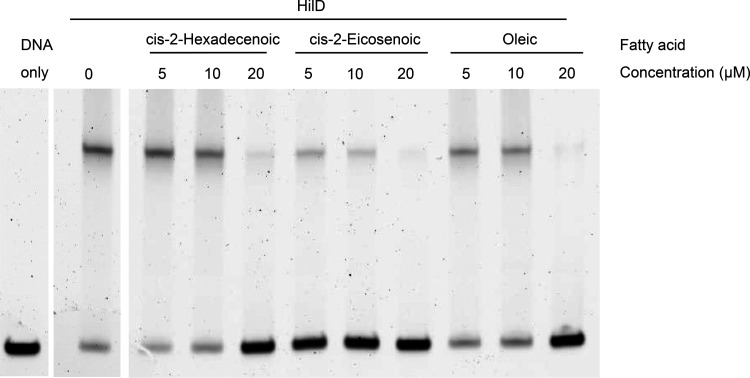
*cis*-2-unsaturated fatty acids inhibit HilD from binding its DNA target. In the presence of 20 μM fatty acid, HilD was completely inhibited from binding *hilC* promoter DNA, while 10 μM did so partially. All wells contained 7.5 nM *hilC* promoter DNA. The indicated lanes contained 50 nM HilD protein. Fatty acids were added at concentrations of 5, 10, and 20 μM as indicated.

The results presented above indicate that *cis*-2-unsaturated fatty acids repress HilD through an inactivation mechanism followed by protein degradation. We hypothesized that these compounds directly interact with HilD, thus impairing its function. HilD binds to the *hilC* promoter, requiring the region from nucleotides −162 to +48 relative to the transcription start site for efficient binding (47). We examined the effects of *cis*-2-unsaturated compounds on the binding of purified HilD to the *hilC* promoter using electrophoretic mobility shift assays (EMSAs). In the absence of DSF, the expected binding of HilD to the *hilC* promoter was demonstrated by the retarded migration of this DNA fragment through the polyacrylamide gel (Fig. 7). Addition of 20 μM c2-HDA impaired HilD’s ability to bind to the *hilC* promoter, whereas 10 μM partially inhibited binding (Fig. 7, left). Similar concentrations of *cis*-2-eicosenoic and oleic acid produced the same pattern of DNA binding inhibition (Fig. 7, middle and right). Therefore, the *cis*-2-unsaturated fatty acids directly inhibit the ability of HilD to interact with its DNA target.

### The DSF c2-HDA represses invasion gene expression in a mouse colitis model.

**FIG 8:**
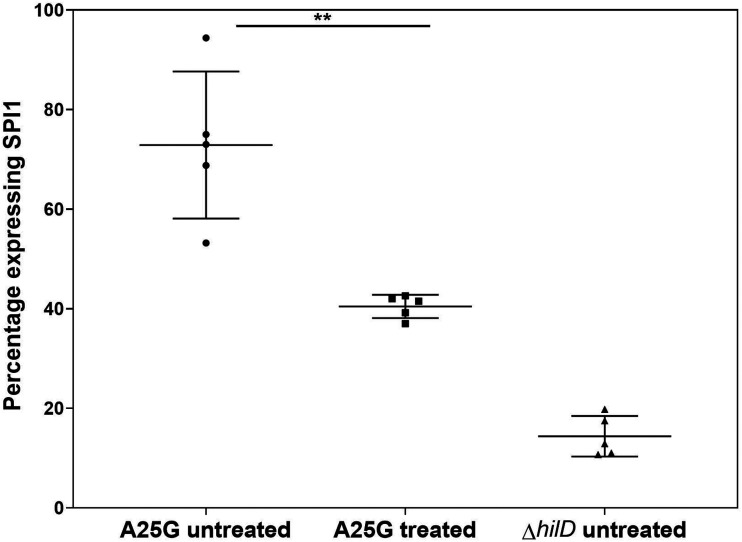
*cis*-2-hexadecenoic acid reduces the percentage of *Salmonella* expressing SPI in the gut. Three groups of mice (*n* = 5/group) were inoculated with *Salmonella* strains carrying *phoN*::*BFP* (for identifying *Salmonella*) and *sicA-GFP* (for monitoring SPI expression), with either a *hilD* UTR A25G mutation or a *hilD* null mutation as shown. Percent SPI1 expression was calculated as the portion of BFP-expressing bacteria that also expressed GFP. Data are presented as percentages with means shown by the horizontal lines and the error bars indicating standard deviations. Asterisks indicate expression levels significantly different from that of the control (**, *P* < 0.01).

We next tested whether c2-HDA would inhibit SPI1-encoded invasion gene expression in the complex chemical environment of the gut. Only a portion of bacteria activate invasion genes in the gut (48, 49). To improve the sensitivity of the assay, we used a strain carrying a *hilD* untranslated region (UTR) A25-to-G (A25G) single base mutation, resulting in increased invasion gene expression due to altered mRNA stability (50). This strain additionally carried a constitutively expressed Δ*phoN*::*BFP* construct for *Salmonella* identification and a *sicA-GFP* reporter fusion to monitor SPI1 expression. The administration of c2-HDA to mice at 1.5 mM in drinking water significantly reduced the percentage of bacteria expressing SPI1 in the cecum (2-fold). The proportion of a Δ*hilD* null mutant expressing SPI1 was 5-fold lower than for the untreated A25G strain, indicating the importance of HilD for invasion activation in the gut (Fig. 8 and Fig. S2). As fatty acids are rapidly absorbed in the upper gastrointestinal tract (51), we presume that small amounts of c2-HDA were available in the cecum. Compared to the *in vitro* potency of c2-HDA (Fig. S3), an estimated concentration of between 2.5 μM and 10 μM would repress SPI1 to the percentage observed in the cecum. Overall, these results demonstrate that the DSF c2-HDA can signal to inhibit invasion gene expression in the gut.

## DISCUSSION

Here we report that *cis*-2-unsaturated fatty acids, typically employed as quorum sensing signals by a range of bacterial species, potently regulate virulence genes in enteric pathogens. In *Salmonella*, c2-HDA interacts with the central SPI1 transcriptional regulator HilD, a member of the AraC family, preventing it from binding its DNA target (Fig. 8). The transcriptional regulators of this family are well known for effector-mediated transcriptional control of metabolic pathways (14). Accumulating evidence that AraC-type transcriptional regulators control virulence has elicited investigation into the environmental signals that they sense (5, 52–54).

In *Salmonella* and other important enteric pathogens, including V. cholerae, AraC-type transcriptional regulators of pathogenicity elements have been reported to sense long-chain fatty acids (9, 20). The animal host secretes bile, containing a mixture of unsaturated fatty acids and surfactants, into the gut lumen for digestion of lipids and protection from pathogens (55). Enteric pathogens, however, have adapted to resist killing by bile and further have integrated bile as a signal of their entry into a host (56). Similarly, they likely use fatty acids as cues for the activation of virulence at the appropriate niche of the gut (9, 17, 41). *cis*-2-unsaturated fatty acids function as quorum sensing signals in *Proteobacteria*, including pathogens of plants and animals, where they signal by regulating c-di-GMP turnover, leading to the regulation of virulence factors (23, 30, 31). In *Salmonella*, however, we demonstrate here a novel mechanism: they interact with the AraC-type transcriptional regulator HilD to control a cascade of invasion genes (Fig. 5 and 7). We propose that c2-HDA binds HilD directly, as has been shown for other fatty acids with ToxT (20). Deactivated HilD is consequently degraded by Lon, reducing the half-life of HilD dramatically.

DSF signaling between species and even kingdoms, resulting in the control of behaviors like biofilm formation, has been reported (26, 27). The ability of specific *cis*-2-unsaturated fatty acids to potently repress HilD raises the question of whether HilD naturally interacts with this class of chemicals in the gut. There are no known dietary sources for *cis*-2-unsaturated fatty acids. It is thus unknown whether *Salmonella* encounters DSFs within an animal host, but it is clear that bacterial species present in the gut produce DSFs. Metagenomic analyses have revealed the existence of the DSF-producing genus *Burkholderia* in wild and laboratory mice (57). Stenotrophomonas maltophilia, which contains a DSF quorum sensing system related to that of *Xanthomonas* (58), is a constituent of the crypt-specific core microbiota of the murine colon, where it is thought to play an important role in crypt protection (59). The DSFs of *Burkholderia* (*cis*-2-dodecenoic acid) and *Stenotrophomonas* (*cis*-11-methyl-2-dodecenoic acid) are less potent in repressing invasion genes than is c2-HDA. Due to the great sensitivity of *Salmonella* to highly specific members of the DSF class, we speculate that this enteric pathogen senses interspecies signals as a cue to its location within the gut and consequently modulates the expression of its virulence determinants.

With the widespread and growing occurrence of antibiotic resistance, remedies aimed at attenuating virulence rather than survival of pathogens would help alleviate selection pressure. Our observations suggest that DSFs provide such an opportunity to be explored for the control of *Salmonella* disease and colonization. c2-HDA is capable of inhibiting SPI1-encoded invasion gene expression at very low concentration and may thus be further investigated as an inhibitor of *Salmonella* infection (Fig. 1). Furthermore, the inactivation of HilD by c2-HDA leading to its rapid degradation is an elegant mechanism for the irreversible deactivation of invasion. Despite the rapid absorption of c2-HDA in the gut, it is likely that a low micromolar amount of this compound would be sufficient to repress invasion gene expression (Fig. 8 and Fig. S3). It might be anticipated that HilD mutants, resistant to the action of c2-HDA, would arise. However, c2-HDA also modulates the activity of the SPI1 alternate AraC transcriptional regulators HilC and RtsA (Fig. 4 and 6), and thus the probability of simultaneous mutations occurring in all three proteins is remote. c2-HDA is therefore a highly potent chemical signal functional in the animal host, which might be exploited in the control of *Salmonella* colonization and possibly that of other enteric pathogens.

## MATERIALS AND METHODS

### Strains.

Salmonella enterica subsp. *enterica* serovar Typhimurium 14028s and Vibrio cholerae C6706 EI Tor, and mutants thereof as listed in Table S1, were used throughout. Deletion mutants were constructed as previously described (60). Briefly, PCR fragments of kanamycin and chloramphenicol resistance genes containing 40-bp homology extensions flanking the gene of interest were generated using plasmids pKD4 and pKD3. The PCR fragments were transformed into a strain expressing λ Red recombinase. Loss of the gene of interest was confirmed using PCR. Unmarked mutants were generated using helper plasmid pCP20 carrying a gene encoding the FLP recombinase. Marked deletions and constructs were transferred using bacteriophage P22 transduction (61).

### Luciferase assays.

Strains carrying *luxCDABE* reporter fusions (Table S1) were grown overnight in LB (containing 5 g of NaCl, 5 g of yeast extract, and 10 g of tryptone) with the necessary antibiotics. Overnight cultures were diluted 100-fold into M9 minimal medium with glucose, antibiotics, and 1 mM nonanoic acid (added to repress SPI invasion gene expression to eliminate background luminescence) and grown overnight. The cultures were washed three times with phosphate-buffered saline (PBS). Bacteria were inoculated at a starting optical density at 600 nm (OD_600_) of 0.02 into 150 μl of LB containing 100 mM morpholinepropanesulfonic acid (MOPS; pH 6.7), the necessary antibiotics, and compounds to be tested in a sealed black-walled 96-well plate. Luminescence was measured every 30 min for 24 h using a BioTek Synergy H1 microplate reader. For V. cholerae luciferase assays, the strain was grown under cholera toxin-inducing conditions (termed AKI) as previously described (62).

### Invasion assay.

Invasion was determined using a gentamicin protection assay as previously described, with modifications (63). Bacteria were grown overnight in LB buffered with 100 mM HEPES (pH 8) in the presence of 20 μM *cis*-2-unsaturated fatty acid compounds. Overnight cultures were washed with PBS, and ∼2 × 10^6^ bacteria were added to 1 ml of HEp-2 cells to maintain a multiplicity of infection of 10. Plates were centrifuged for 10 min at 100 × *g* and incubated for 1 h at 37°C. Plates were then washed and gentamicin was added at a concentration of 20 μg/ml to the media. After 1 h of incubation, cells were washed and lysed with 1% Triton X-100. Lysates were plated on agar plates, and recovered intracellular bacteria were counted. Percent invasion in the presence of *cis*-2-unsaturated fatty acid compounds was calculated by comparison with the untreated cultures.

### Methyl ester synthesis.

Esters of c2-HDA and *cis*-2-eicosenoic acid were prepared by reacting methanolic acid with the compounds. The reaction mixture was refluxed at 80°C for 30 min. Thin-layer chromatography (TLC) was employed to monitor the progress of the esterification reaction, using ethyl acetate in hexane as the mobile phase. Phosphomolybdic acid was used to visualize product formation with gentle heating. The solvent was evaporated and the product lyophilized overnight before use.

### Half-life assay.

HilD half-life assays were performed as previously described (7, 17). Briefly, we utilized a strain with *hilD* under the control of a promoter (P*_tetRA_*) and a C-terminal 3×FLAG tag construct. Cultures were grown overnight and then diluted 1:100 into LB containing 100 mM MOPS (pH 6.7), 1 μg/ml of tetracycline (for P*_tetRA_* induction), and 20 μM fatty acid compounds to be tested. After 2.5 h of growth, OD was adjusted to 1 for all cultures. Transcription and translation were halted by adding a cocktail of antibiotics. Cultures were incubated at 37°C, and samples were taken every 30 min for Western blot analysis using an anti-FLAG antibody. The HilD-3×FLAG signal was quantified by detecting the density of bands using UVP LS software (UVP LLC). Half-life was calculated as the difference in density between time point zero and the last signal time point, as previously described (7).

### HilD expression and purification.

*hilD* was amplified and cloned into pCAV4, a modified T7 expression vector that introduces an N-terminal 6×His-NusA tag followed by a human rhinovirus (HRV) 3C protease site. The construct was transformed into E. coli BL21(DE3). The expression strain was grown at 37°C in terrific broth to OD_600_ of 1 and induced with 0.3 mM isopropyl-β-d-thiogalactopyranoside (IPTG). Induced cultures were grown overnight at 19°C. Cells were pelleted and resuspended in nickel buffer (20 mM HEPES [pH 7.5], 500 mM NaCl, 5% glycerol, 30 mM imidazole, and 5 mM β-mercaptoethanol). Cells were lysed by sonication, and insoluble cell debris was removed by centrifugation at 13,000 rpm. The clarified supernatant was applied to a 5-ml chelating HiTrap (GE) charged with nickel sulfate. The column was washed with nickel buffer, and the protein was eluted with a 30 mM to 500 mM imidazole gradient. The pooled eluates were dialyzed overnight into heparin buffer (20 mM HEPES [pH 7.5], 300 mM NaCl, 1 mM EDTA, 5% glycerol, and 1 mM dithiothreitol [DTT]) in the presence of HRV 3C protease to remove the 6×His-NusA tag. Following dialysis, the protein was applied to a 5-ml heparin HiTrap (GE), washed with heparin buffer, and eluted with a gradient of 300 mM to 1 M NaCl. HilD was then concentrated and injected onto a Superdex 200 10/300 sizing column (GE) equilibrated in HilD storage buffer (20 mM HEPES [pH 7.3], 500 mM KCl, and 1 mM DTT). The final concentration of purified HilD was 10 to 20 mg/ml.

### EMSAs.

Electrophoretic mobility shift assays (EMSAs) were performed as previously described (9). Briefly, 7.5 nM *hilC* promoter DNA (nucleotides −162 to +48 [45]) was mixed with 50 nM HilD in a binding buffer containing 20 mM KCl, 1% glycerol, 1 mM DTT, 0.04 mM EDTA, 0.05% Tergitol NP-40, and 20 mM HEPES (pH 7.3). *cis*-2-unsaturated fatty acid compounds were tested at concentrations of 5, 10, and 20 μM. As a control, oleic acid was also tested at concentrations similar to those of the *cis*-2-unsaturated fatty acids. Binding was performed at room temperature for 20 min. Samples were separated on 6% Novex Tris-borate-EDTA (TBE) DNA retardation gels, and DNA was stained using SYBR green (Invitrogen).

### Animal experiments.

Female C57BL/6 mice, 6 to 7 weeks old, were provided with c2-HDA at a concentration of 1.5 mM, or the vehicle control (Solutol HS 15), as their sole drinking water source throughout the experiment. Mice were inoculated by gastric gavage with 20 mg of streptomycin 24 h after the introduction of treated water. Bacterial strains were grown overnight in M9 minimal medium supplemented with 0.2% glucose. Cultures were washed twice and resuspended in PBS. Mice were inoculated with ∼10^8^ bacteria by gastric gavage 24 h after treatment with streptomycin (64). Mice were euthanized 1 day after *Salmonella* infection using carbon dioxide according to American Veterinary Medical Association guidelines, and cecal contents were collected.

### Flow cytometry.

Cecal contents were diluted into 5 ml of PBS, vortexed for 2 min, and filtered with 5-μm filters to remove debris. Recovered cells were pelleted and resuspended in 1 ml of 4% paraformaldehyde in 1× PBS. Cells were fixed for 30 min at 4°C, pelleted to remove paraformaldehyde, and resuspended in PBS. Flow cytometry was performed as previously described (33). Recovered cells were analyzed for blue fluorescent protein (BFP) and green fluorescent protein (GFP) expression using an Attune analyzer NxT flow cytometer (Thermo Fisher). *Salmonella* was identified by BFP expression, and GFP was used to monitor SPI1 expression. Data were analyzed using FlowJo 10.6.1 software (FlowJo LLC).

### Statistical analysis.

Means of treated and untreated samples were compared using Student’s *t* test.

### Ethics statement.

Animal studies were approved by the Institutional Animal Care and Use Committee at Cornell University (protocol 2012-0074).

## Supplementary Material

Supplemental file 1
