# Supplementary material for: Diffusible Signal Factors Act through AraC-Type Transcriptional Regulators as Chemical Cues To Repress Virulence of Enteric Pathogens
Source: Infect Immun. 2020 Sep 18;88(10):e00226-20. doi: 10.1128/IAI.00226-20 (PMC7504960; doi:10.1128/IAI.00226-20)

Supplementary information

Supplementary table 1. Strains and plasmids

Supplementary figure 1. A) Regulation of *Salmonella* pathogenicity island 1-encoded type III secretion system by AraC-type transcriptional regulators. HilD forms a feedforward loop together with RtsA and HilC, all AraC-type regulators, to activate *hilA*. HilA induces the type III needle complex and effector protein genes. Short chain fatty acids (SCFM), long chain fatty acids (LCFA), and diffusible signal factors (DSFs) repress HilD post-transcriptionally. B) cis-2-hexadecenoic acid has no effects on bacterial growth. Cultures were grown in the presence of 20 µM cis-2-hexadecenoic acid and oleic acid. The control cultures contained the vehicle only (DMSO for cis-2-hexadecenoic acid and cis-2-eicosenoic acid, and ethanol for oleic acid) at identical concentration to the treated culture.

Supplementary figure 2. Flow cytometry histograms for cecal bacteria. Mice were inoculated with strains carrying constitutively expressed BFP (Δ*phoN::BFP*) and a *sicA-GFP* reporter fusion, with either an A25G or Δ*hilD* null mutation. The peaks to the right represent the portion of BFP population that is expressing GFP. Gating controls were grown in LB overnight for use in setting gates.

Supplementary Figure 3. Micromolar-range concentration of cis-2-hexadecenoic significantly reduce the proportion of bacteria expressing SPI1. A) The strains

carrying a constitutively expressed BFP (Δ*phoN::BFP*) and a *sicA-GFP* reporter fusion, with either an A25G or a Δ*hilD* null mutation, used for the *in vivo* experiments were grown *in vitro* under SPI1 inducing conditions. The A25G mutant was treated with varying concentrations of cis-2-hexadecenoic acid as shown. Percentage of bacteria expressing SPI1 was determined by flow cytometry. The control cultures contained the vehicle only at identical concentration to the chemical-containing cultures. Error bars represent standard deviations of 3 replicates. B) Flow cytometry histograms. Peaks to the right represent the percentage of bacteria expressing SPI1.

Supplementary table 1

Strain	Genotype	Reference
CA4622	Δ*fadE, philA::luxCDABE*	This study
CA4600	Δ*fadL, philA::luxCDABE*	This study
CA4580	Δ*hilE, psopB::luxCDABE*	This study
CA4579	Δ*lon*, *psopB::luxCDABE*	This study
CA4578	Δ*hilD*, *psopB::luxCDABE*	This study
CA4577	Δ*fliZ*, *psopB::luxCDABE*	This study
CA4576	Δ*hilA*, *psopB::luxCDABE*	This study
CA4575	Δ*rtsA*, *psopB::luxCDABE*	This study
CA4574	Δ*hilC*, *psopB::luxCDABE*	This study
CA4834	*tetRA→rtsA, ΔhilD, psopB::luxCDABE*	This study
CA4526	*tetRA→hilD, ΔrtsA, ΔhilC*, *philA::luxCDABE*	This study
CA2446	*tetRA→hilD-3xFLAG attλ:pDX1::hilA'-lacZ*	[16]
CA3017	*tetRA→hilD-3xFLAG attλ:pDX1::hilA'-lacZ, Δlon*	[16]
CA4526	*tetRA→rtsA, ΔhilC*, *ΔhilD*, *philA::luxCDABE*	This study
CA4693	*tetRA→hilC*, *ΔrtsA*, *ΔhilD*, *philA::luxCDABE*	This study
CA4993	*A25G hilD, phoN::BFP, sicA→GFP, malXY::cam*	This study
CA5007	Δ*hilD*, *malXY*::cam	This study

**Plasmids**		
pBA426	*hilA::luxCDABE*	[16]
pBA409	*sopB::luxCDABE*	[16]
pCA239	*ctxAB::lux*	This study
pCA240	pCAV4-*hilD*	This study

Supplementary figure 1.

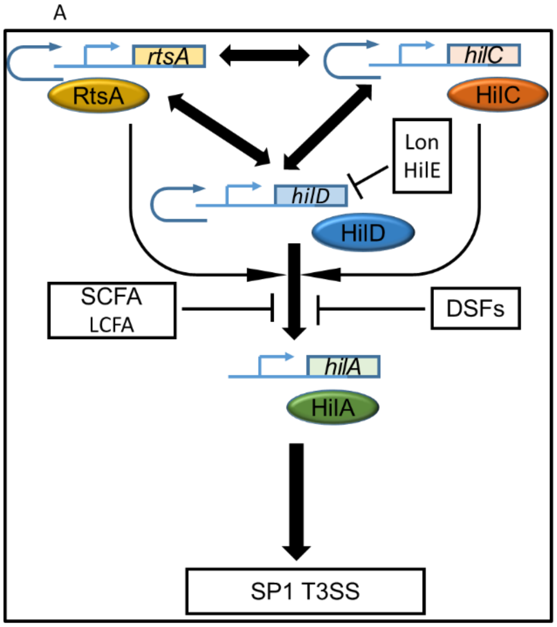

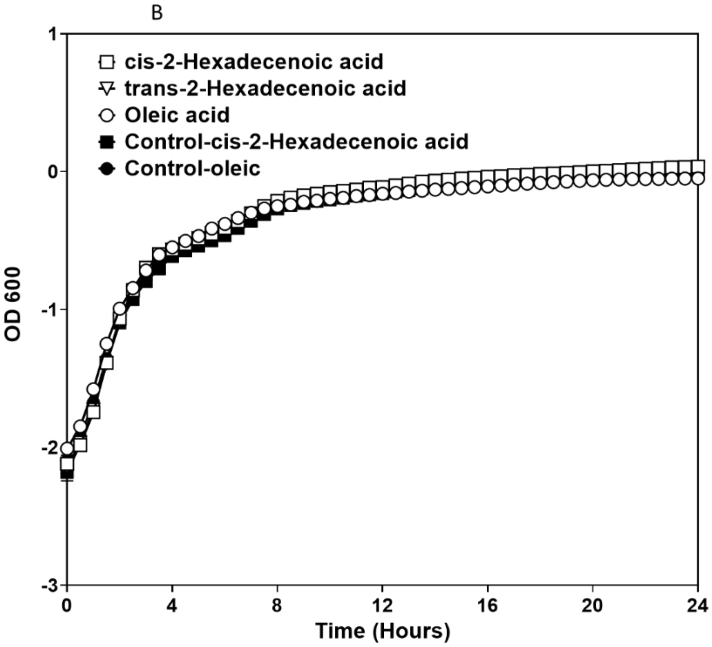

Supplementary figure 2.

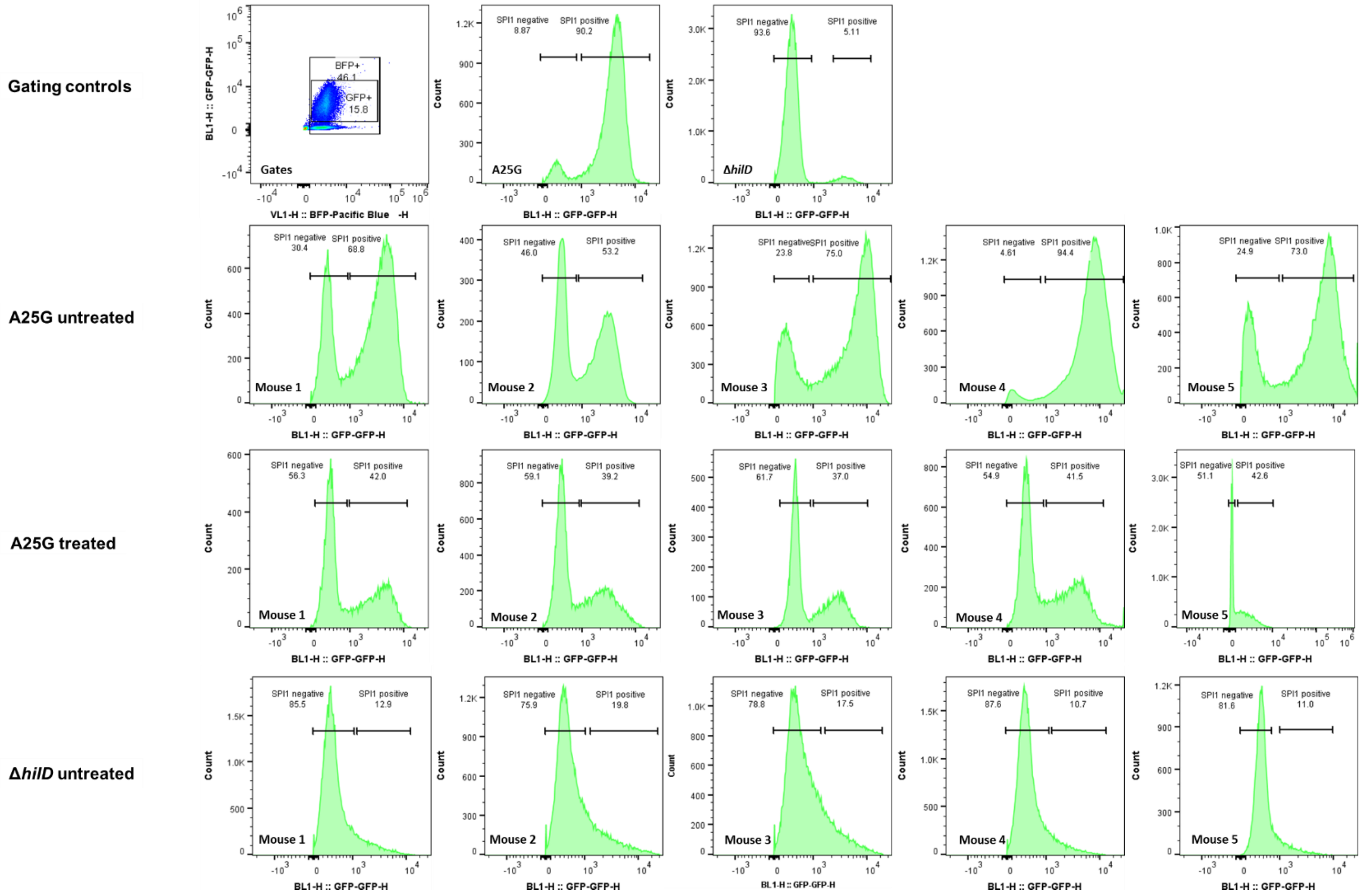

Supplementary Figure 3.

Figure A and B

A

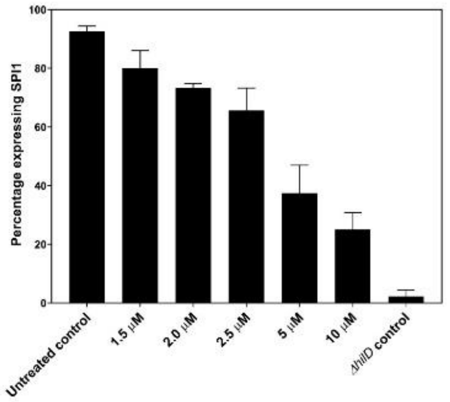

B

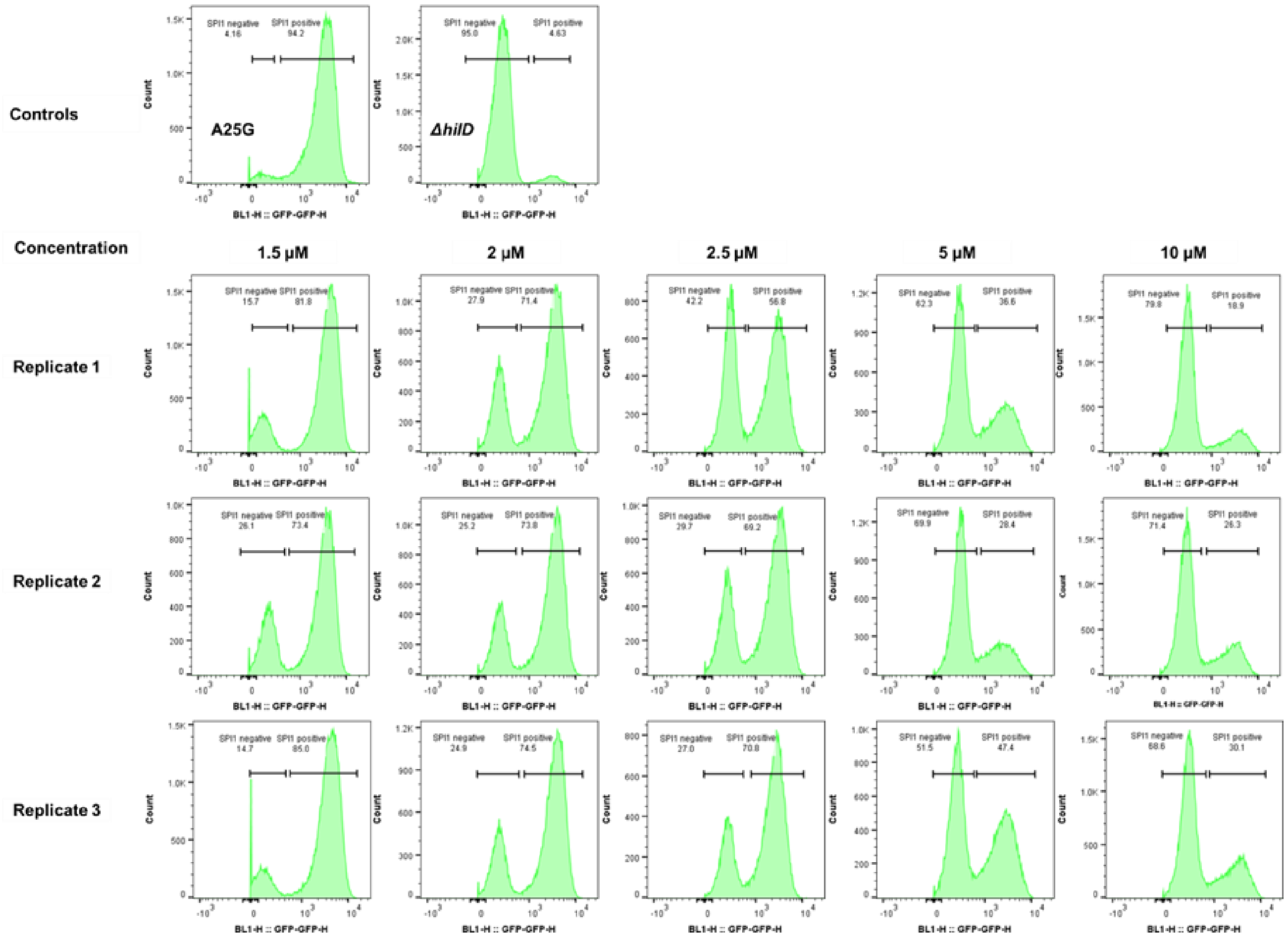

Supplement: Supplemental file 1 [file IAI.00226-20-s0001.pdf]
